# A Clinical-Psychological Perspective on Somatization Among Immigrants: A Systematic Review

**DOI:** 10.3389/fpsyg.2018.02792

**Published:** 2019-01-17

**Authors:** Roberta Lanzara, Mattia Scipioni, Chiara Conti

**Affiliations:** Department of Psychological, Health and Territorial Sciences, Università degli Studi G. d'Annunzio Chieti e Pescara, Chieti, Italy

**Keywords:** immigrants, immigration, somatization, somatic symptoms, traumatic experience

## Abstract

**Background:** Somatic and psychopathological conditions (e.g., anxiety, depression, post-traumatic stress disorder, and somatization) are frequent among immigrants belonging to various ethnic groups. Worldwide findings on the epidemiology regarding specific mental conditions still vary with respect to different migration samples and migration contexts. This inconsistency also holds true in the incidence of somatization among migrants. We carried out a systematic review analyzing the relationship between migration and somatization by providing a qualitative data synthesis of original research articles on the topic.

**Methods:** According to PRISMA guidelines, we conducted a systematic search of the literature on PubMed, Scopus, ISI Web of Science, PsycINFO, Google Scholar, and ScienceDirect. The articles were selected using multiple combinations of relevant search terms (e.g., defined somatization and related disorders, and migration status). Each database was searched systematically from January 2000 to December 2017.

**Results:** The initial search identified 338 records, of which 42 research reports met the predefined inclusion criteria and were analyzed. Most studies (*n* = 38; 90%) were cross-sectional. The main findings of this study are that migrants with somatization exhibited more psychological distress, had an increased perceived need for healthcare service utilization, and reported more post-migration living difficulties and/or post-traumatic stress disorder than those without somatization. It was also found that specific individual features mediate the association between somatization and migration. The prevalence and correlates of somatization were found to vary across the immigrant groups, depending on cultural variation in reasons for migration, stress exposure, explanatory models of illness, coping, and other individual variables.

**Conclusion:** Somatization is a challenge for health professionals due to its vague nature. In this regard, clinical management of immigrant patients should include further efforts to address emotional distress, with special attention to social, cultural, and linguistic differences.

## Introduction

Migration can be defined as “a process of moving, either across an international border, or within a State. Encompassing any kind of movement of people, whatever its length, composition and causes; it includes refugees, displaced persons, uprooted people, and economic migrants” (Perruchoud, 2004). During the period from 2000 to 2017, the total number of international migrants increased from 173 to 258 million persons, an increase of 85 million (49%); 65 million of the world's internal and international migrants are forcibly displaced today (United Nations Population Division Department of Economic Social Affairs, 2009, 2017). Most migration processes may be conceptualized as a series of mainly stressful life events, each with the cumulative capacity to increase the risk for a broad range of mental health problems (Carta et al., 2005). Immigrants are often subjected to specific risk factors related mainly to exposure to stressful and traumatizing experiences (Shiroma and Alarcon, 2011; Rohlof et al., 2014), including the migrant status itself and, further, the associated acculturative stress and adaptation process to a new culture, racial discrimination, urban violence, abuse by law enforcement officers, and forced removal or separation from their families (Bermejo et al., 2010; Bragazzi et al., 2014). Several studies indicate that the incidence of psychological distress (Carta et al., 2005), post-traumatic stress disorder (PTSD) (Silove et al., 1998), major depressive disorder (Beirens and Fontaine, 2011), and somatization in diverse ethnic immigrant groups has increased all over the world (Haller et al., 2015).

Somatization is a complicated concept to define. Straddling the interface between physical and psychological ill health, it is often viewed from a range of different perspectives (Gureje et al., 1997). One finds in the literature definitions emphasizing the presence of multiple complaints in diverse areas of the body (Mai and Merkey, 1980; Escobar et al., 1989), formulations in which fear of having a serious physical disorder in the absence of supporting physiologic impairments is stressed (Barsky and Klerman, 1983), and others in which physical complaints are seen as manifestations of hidden psychiatric morbidity (Bridges and Goldberg, 1987). The concept of somatization has its origins in the work of Freud (Breuer and Freud, 1893–1895), who proposed the idea of conversion as a main defense mechanism. Following that, Alexander (Alexander, 1950) brought the notion of emotional equivalents, also proposed by Freud, into the concept of vegetative neurosis and psychosomatic diseases. Of late, somatization is often regarded as “a tendency to experience and communicate somatic distress in response to psychosocial stress and to seek medical help for it” (Lipowski, 1988). Somatization is most often associated with depressive and anxiety disorders (Simon et al., 1999; Haller et al., 2015); its persistent form is especially costly and difficult to prevent and manage. It thus poses major medical, social, and economic challenges (Lipowski, 1988).

Physical manifestations implicated in somatization can be aligned across a spectrum of numerosity, severity, and functional impairment, extending from just one or a few transient symptoms at one end, to having multiple severe symptoms for a long period of time and therefore meeting diagnostic criteria for somatoform disorder (SD) according to the Diagnostic and Statistical Manual of Mental Disorders (4th ed.; DSM-IV) (American Psychiatric Association, 1994) or somatic symptom disorder (SSD) according to the 5th edition (DSM-5) (American Psychiatric Association, 2013), at the other end (Jackson and Kroenke, 2008).

In the most recently released DSM-5, the conceptualization of somatization and what was previously termed somatoform disorder has changed substantially compared with previous diagnostic systems. This reflects an effort to overcome the limitations of the DSM-IV definition, which was organized centrally around the concept of medically unexplained symptoms (MUS). The current diagnostic criteria for SSD requires the presence of somatic symptoms combined with a substantial impact of these symptoms on thoughts, emotions, and behaviors (Carta et al., 2005), by emphasizing the extent to which feelings concerning their somatic symptoms are disproportionate or excessive. Somatization is a psychological dimension common to all people; it manifests in response to psychosocial stress brought about by life events and situations that are personally stressful to the individual (Lipowski, 1988). It has been strongly associated with the migrant status itself, as a coping or adapting mechanism extremely disadvantageous for health (Castillo et al., 1995; Escobar, 1995; Kirmayer and Sartorius, 2007; Radl-Karimi et al., 2018).

There are major differences in somatization among immigrants according to the moderating effects of psychosocial features such as their ethnic, cultural and religious background; exposure to trauma; reasons for migration; and other individual differences (Kirmayer and Young, 1998). These characteristics also differentially affect illness perception, communication of symptoms, and help-seeking behavior. Clinical-psychological assessment and treatment of somatization thus can be particularly challenging in multicultural contexts (Carta et al., 2005), imposing a considerable economic burden on health services.

To our knowledge, systematic attempts to investigate the frequency and clinical-psychological correlates of somatization in a wide spectrum of migrant populations have thus far not been undertaken. To address these gaps, we carried out a systematic review examining the prevalence, clinical manifestation, etiology, and treatment of somatization in individuals with migratory background, by providing a qualitative data synthesis of the studies. The inclusion of papers in this review was extended to those investigating somatization as the somatic clinical presentation of psychological distress, high levels of somatic preoccupation, MUS, or mental disorder (according to DSM-IV or DSM-5).

Based on the extant literature, we expected that: (1) somatization would be significantly associated with migration because of the supposed high exposure to stressful experiences in individuals with migratory backgrounds; and (2) the prevalence and correlates of somatization would vary across immigrant groups, depending on cultural variations in reasons for migration, trauma exposure, coping, and other individual variables.

## Materials and Methods

### Eligibility Criteria

Eligible articles included all English language papers published in peer-reviewed journals from January 2000 to December 2017, reporting data on the presence of somatization in first-generation immigrants. When a title or abstract seemed to describe a study eligible for inclusion, the full text was examined to consider its relevance according to the inclusion criteria. Reviews, meta-analyses, commentaries, letters to the editor, books or book chapters, abstracts, and clearly irrelevant papers were excluded. Since somatization is particularly difficult to operationalize, we also excluded articles published before 2000 for a greater homogeneity on the meaning of somatization.

The included studies had to:

1. examine a population of first-generation adult immigrants. Immigrants are defined as foreign born people who have moved to another country for the purpose of settlement (Perruchoud and Redpath-Cross, 2011). This definition includes economic migrants, temporary foreign workers, foreign students, documented and undocumented migrants, refugees, and asylum seekers;
2. investigate somatization defined as somatic presentation of psychological distress, or MUS, or high levels of somatic preoccupation and worry about illness, or the clinical presentation of psychiatric disorder according to DSM-IV or DSM-5;
3. use questionnaires, subscales, semi-structured interviews, or DSM criteria for assessing somatization.

### Information Sources and Searches

This systematic review was conducted according to the Preferred Reporting Items for Systematic Reviews and Meta-Analyses (PRISMA) guidelines (Liberati et al., 2009). PubMed, Scopus, ScienceDirect, ISI Web of Science, PsycINFO, and Google Scholar databases were systematically searched in November 2017 using the following Boolean string: (“immigrant^*^” OR “migrant^*^” OR “immigration” OR “migration” OR “refugee^*^” OR “asylum seeker^*^”) AND (“somatization” OR “somatizer^*^” OR “medically unexplained symptom^*^” OR “functional disease” OR “functional symptom^*^” OR “somatic symptom^*^ disorder” OR “illness anxiety disorder” OR “conversion disorder” OR “functional neurological symptom^*^” OR “psychological factors affecting med^*^” OR “factitious disorder”) [All Fields]. Each database was systematically searched for articles from January 2000 to December 2017. After performing the initial search, duplicates were identified and discarded. Titles and abstracts were screened and, for reports thus identified as potentially relevant, full texts were checked for eligibility. Studies were discarded where the full text was unavailable. Searching and determining the eligibility of target responses were carried out independently by the three investigators.

### Selection of Articles and Data Extraction

Two of the authors (R.L., M.S.) performed the initial data extraction by removing duplicates and all the articles that appeared clearly irrelevant based on the salience of the title and after reading the specific abstract. The full texts of the remaining studies were independently assessed for eligibility by all authors. After a full-text evaluation of the potentially relevant studies, the three authors reached a consensus regarding eligibility and excluded all the research articles that not meet the inclusion criteria.

### Analysis and Data Synthesis

The methods described here fulfilled the PRISMA guidelines (Liberati et al., 2009), as a meta-analysis was deemed inappropriate due to the heterogeneity of the examined study designs. To assess the risk of bias, and working independently; the reviewers each determined the adequacy of the methodology in terms of reliability. Within the sample selected for review, studies were categorized by summarizing and comparing significant information and specifying the measures of the assessed variables for each (see Table 1 for a detailed description of the reviewed studies).

**Table 1:** Distribution of the 43 relevant selected studies, including the reference, the population target, the aims, the measures of somatization, and the main results of the investigation.

**Reference**	**Aims**	**Population target and geographic location**	**Country of origin**	**Measures of somatization**	**Results**
Aragona et al., 2010	To explore the relationship between somatization and self-reported traumatic experiences and post-traumatic symptoms in patients attending a primary care service for immigrants.	*N* = 101 ♀ ♂ Age = 19–67 Primary care outpatients Italy	Europe, Asia, South America, Africa	- BSI-21*	Somatization prevalence: 38.6%; Somatization + traumatic experiences: 69.2%.
Aragona et al., 2008	To investigate the effect of gender and marital status on somatization in immigrants of four ethnic groups.	*N* = 301 ♀ ♂ Age (*M*) = 35.85 Primary care outpatients Italy	Europe, Asia, South/Central America, Africa	- BSI-21*	Somatization prevalence: female > male; married > unmarried; Caucasians and South-Central Americans > other ethnic groups.
Aragona et al., 2011	To evaluate the role of post-migration living difficulties (PMLD) on somatization.	*N* = 101 ♀ ♂ Age = 19–67 Primary care outpatients Italy	Europe, Asia, South America, Africa	- BSI-21*	Somatization prevalence: 38.6% Somatization + traumatic experiences: 69.2% Somatization + PTSD: 30.7% PLMD: somatizers > non-somatizers.
Aragona et al., 2013	To study potentially traumatic events, PTSD, anxiety, depression, somatization and PMLD in primary care immigrants.	*N* = 391 ♀ ♂ Age = 18–79 Primary care outpatients Italy	Europe, Asia, South America, Africa	- BSI-21*	Somatization + PTSD: 80% Somatization + No PTSD: 23.1% No somatization + PTSD: 20% No somatization + No PTSD: 76.9%.
Aragona et al., 2012	To study somatization in a large sample of immigrants.	*N* = 3,051 ♀ ♂ Age = 17–86 Primary care outpatients Italy	Europe, Asia, South America, Africa	- BSI-21*	Somatization prevalence: 25.6%; female > male; older > younger; South Americans > other ethnic groups.
Aragona et al., 2005	To investigate the current prevalence of somatization and to evaluate the comparative rates of somatic complaints in immigrants of four ethnic groups.	*N* = 301 ♀ ♂ Age = 16–70 Primary care outpatients Italy	Europe, Asia, South/Center America, Africa	- BSI-21*	Somatization prevalence: 35.2%; female > male; South Americans > other ethnic groups.
Bäärnhielm and Ekblad, 2000	To explore structures of illness meaning among somatizing Turkish-born migrant women.	*N* = 10 ♀ Age = 31–48 Psychiatric or primary care outpatients Sweden	Turkey	- SCID-RV- Medical records	All participants experienced and communicated psychological distress in the form of physical symptoms.
Beirens and Fontaine, 2011	To investigate the combination of two cultural explanations (somatization vs. psychologization and emotion mediation) with two acculturative explanations (acculturative stress vs. acculturative transition) to explain these differences.	*N* = 719 ♀ ♂ Age = 18–58 Turkish immigrants, Turkish majority members, Belgian majority members Belgium	Turkey	- Not standardized interview	Somatization prevalence: Turkish majority members > Turkish immigrants > Belgian majority members.
Borra, 2011	To explore Turkish women's idioms of distress; to contribute to the development of a valid and reliable diagnostic technique for depressive disorder in Turkish women.	*N* = 20 ♀ Age = 20–50 Depressed outpatients Netherlands	Turkey	- Not standardized interview	Distress: somatizers > non-somatizers.
Bragazzi et al., 2014	To investigate differences in the somatic perception between immigrants and Italians and between two groups of immigrants living in Italy.	*N* = 329 ♀ ♂ Age (*M*): 35.0 (Immigrants); 38.3(Italians) Clinic outpatients Italy	South America, Africa	- MSPQ*	Somatization: female > male; no immigrant group differences (*p* = 0.18); immigrant groups > Italian group.
Choi et al., 2017	To examine the relationship between trauma, psychiatric symptoms and life satisfaction of North Korean refugees resettled in South Korea.	*N* = 211 ♀ ♂ Age: (*M, SD*) = 38.49, ±12.22 North Korean refugees Republic of Korea	North Korea	- SCL-90-R	Somatization + previous traumatic events: positive correlation (*r* = 0.33, *p* < 0.001). Somatization + economic satisfaction: negative correlation (*r* = −0.19, *p* < 0.01).
Cwikel et al., 2008	To examine the prevalence and correlates of a full range of mental health diagnoses in primary care clinics in Israel.	*N* = 976 ♀ ♂ Age = 25–75 Primary care patients Israel	Various ethnic groups	- SCL-90-R	Somatization: female > male; no ethnic group differences.
David et al., 2012	To evaluate the effect of migration on psychosocial state of hyperemesis gravidarum patients.	*N* = 753 ♀Age = 15–44 Women treated for hyperemesis gravidarum Germany	Turkey, Southwest Asia, Northeast Africa, Ex-Yugoslavia	- SCL-90-R*	Hyperemesis gravidarum: Immigrant patients > native patients; Somatization: no group differences.
Deisenhammer et al., 2012	To study the impact of both ethnicity and migration on the manifestation of depression.	*N* = 136 ♀ Age = 18–76 Depressed patients: Austrian-origin, Turkish immigrants, Turkish living in Turkey Austria	Turkey	- BSI-21*	Somatic symptoms: Turkish immigrants > Turkish living in Turkey > Austian-origin.
Dreher et al., 2017	To compare Vietnamese and German patients regarding cultural dynamics of symptom presentation upon first admission to a psychiatric outpatient service	*N* = 219 ♀ ♂ Age = 17–65 Psychiatric outpatients:Vietnamese immigrants, German-origin Germany	Vietnam	- PHQ-15*	Severe somatization rates: Vietnamese patients (32.7%), German patients (12.8%).
Fenta et al., 2006	To examine the mental health service utilization patterns of Ethiopians in Toronto.	*N* = 342 ♀ ♂ Age = 18–59 Immigrant outpatients Canada	Ethiopia	- DIS	Somatic symptoms prevalence: Ethiopian patients (63.2%); female > male; Healthcare service use: somatizers > non-somatizers.
Heredia Montesinos et al., 2012	To analyze the interrelationship of stigma, depression, overall psychological distress, and somatic symptoms.	*N* = 63 ♀ Age = 28–72 Depressed patients Germany	Turkey	- SCL-90-R- SOMS-II	Positive association between depression, psychological distress, and somatic symptoms.
Hondius et al., 2000	To analyze the relative contribution of different forms of violence, demographic, and asylum variables to the health complaints of refugees.	*N* = 636 ♀ ♂ Primary care outpatients Netherlands	South/Central America, Western Asia, Turkey, Iran	- Not standardized interview	Positive association between violent events, post-migration living difficulties, and somatic complaints.
Karasz et al., 2007	To examine evidence for several theoretical processes shaping the relationship between culture and illness experience.	*N* = 73 ♀ ♂ Age = 19–62 European and Asian immigrants living in US US	South Asia, Europe America	- Not standardized interview	Somatization prevalence: no group differences. 20% of EAs psychological problems (but none of SAs) were explained entirely by somatic causes; 43% of EAs psychological problems included at least one physical cause; 4% of SAs psychological problems included at least one physical cause.
Mak and Zane, 2004	The phenomenon of somatization was explored in relation to the experiences of acculturation, stress, support, and distress.	*N* = 1,747 Age = 18–65 Community sample US	China	- SCL-90-R- SSI	Somatization: 57.2% on SCL-90-R; 12.9% on SSI; female > male; older > younger; low education level > high education level.
Mendoza et al., 2017	To evaluate the role the role of migration stressors and social support on poor mental health among Filipino female domestic workers.	*N* = 261 ♀ Age = 18–64 Domestic workers China	Filipino	- PHQ-15*	Positive association between somatization, symptoms severity, and post-migration stress (*p* < 0.01). No association between social support and somatization.
Miranda et al., 2005	To examine the prevalence of depression, somatization, alcohol use and drug use among black American women.	*N* = 9,151 ♀ Age < 43 US-origin, African-origin, and Caribbean-origin US	America, Africa, Caribbean	- PRIME-MD	Somatization: no group differences.
Mirdal, 2006	To study whether and how the changes that had taken place in actual living conditions would be reflected in the women's subjective perception of their health condition.	*N* = 46 ♀ Age >31 Community sample Denmark	Turkey	- Not standardized interview	Although the living condition of the women had improved and the number of somatic complaints had decreased, the level of distress was still high 20 years later.
Mölsä et al., 2014	To investigate mental and somatic health, and to evaluate the role of pre-migration trauma and post-migration stressors among the refugees.	*N* = 256 ♀ ♂ Age = 50–80 Somali immigrants, Finnish controls Finland	Somalia	- SCL-90-R	Somatization: no group differences. High levels of pre-migration traumatic events were associated with high levels of somatization symptoms (*p* < 0.01).
Mölsä et al., 2017	To analyze healthcare services utilization patterns of older immigrants in Finland, and to investigate the presence of somatization in older Somali refugees and pair-matched Finnish controls.	*N* = 256 ♀ ♂ Age = 50–80 Somali immigrants, Finnish controls Finland	Somalia	- SCL-90-R	Somatization: no group differences.
Morawa et al., 2017	To analyze variations in the severity of somatization according to sociodemographic and migration-related variables.	*N* = 335 ♀ ♂ Age = 20–69 Community sample Germany	Turkey	- PHQ-15*	Somatization prevalence: 24.2%; female > male; first generation immigrants > second generation immigrants; lower language proficiency > higher language proficiency; Severe somatization + severe depression: 53.1%.
Nadeem et al., 2008	To examine the relations between sociodemographic characteristics, stigma, depression, somatization, and treatment preferences.	*N* = 1,893 ♀ Age (*M*; *SD*) = 29.1, ±8.6 Immigrant and US-born outpatients with mental health problems US	America, Africa, Caribbean	- DSM-IV	Somatization: no group differences.
Nadeem et al., 2009	To investigate the differences in treatment preferences and to examine perceived need for care for mental health problems as a possible contributor to ethnic disparities in receiving care.	*N* = 1,577 ♀ Age (*M*; *SD*) = 28.9, ±8.5 Immigrant and US-born depressed outpatients US	American, Africa, Caribbean	- DSM-IV	Somatic complaints: 67% Somatization: no group differences. >1 Somatic symptoms: < Perceived need for mental health.
Nickel et al., 2006	To examine whether bioenergetic exercises significantly influence the inpatient psychotherapeutic treatment results for Turkish immigrants with chronic somatoform disorders.	*N* = 128 ♀ ♂ Age >18 Immigrants with chronic somatoform disorders Germany/Austria	Turkey	- SCL-90-R*	Bioenergetic exercises improved somatization.
Perron and Hudelson, 2006	To study how asylum seeker and refugee patients who were identified by their physicians as somatizing make sense of their suffering.	*N* = 26 ♀ ♂ Age (*M*) = 36 General medicine outpatients Switzerland	Ex-Yugoslavia	- Not standardized interview- Medical records	Patients attributed the onset of somatic symptoms to past traumatic experiences and tended to attribute their persistence to current living conditions and uncertain legal status. Patients formulated their suffering in both medical and social/legal terms and sought help from physicians for both types of problems.
Rask et al., 2015	To examine the association between mental health symptoms and mobility limitation in migrants.	*N* = 2,249 ♀ ♂ Age = 18–64 Russian, Somali and Kurdish migrants Finland	Russia, Somalia, Kurdistan	- SCL-90-R*	Mobility limitation: somatizers > non-somatizers.
Rask et al., 2016	To assess the prevalence of mental health symptoms in Russian, Somali and Kurdish origin migrants in Finland.	*N* = 2,322 ♀ ♂ Age = 18–64 Russian, Somali and Kurdish migrants Finland	Russia, Somalia, Kurdistan	- SCL-90-R*	Somatization prevalence: Kurdish (28.9%); Russian (14.8%); Somali (12.9%). Somatization: female > male.
Ritsner et al., 2000	To examine somatic distress in an immigrant population in Israel, to explore its relationship with psychological distress symptoms and health care-seeking behavior, and to determine its correlation with the length of residence in Israel.	*N* = 966 ♀ ♂ Age = 18–87 Russian-born Jewish Israel	Russia	- BSI*	Somatization prevalence: 21.9%; older > younger; divorced/widowed > others; Number of somatic symptoms: female > male; high length of residence > low length of residence. Distress + somatization prevalence: 20.4%. Somatization + psychological distress: positive correlation; Somatization + help-seeking behavior: positive correlation.
Sachs et al., 2008	To explore the experiences, coping strategies, and psychological distress of Tibetan refugees who reported trauma exposure.	*N* = 769 ♀ ♂ Age = 16–78 Tibetan refugees India	Tibet	- SCL-90-R*	Low somatization scores on SCL-90-R (*M* = 1.28, *SD* = 0.30).
Salinero-Fort et al., 2015	To estimate and compare the prevalence of the most common mental disorders between Latin American-born and Spanish-born patients.	*N* = 1,594 ♀ ♂ Age = 18–55 Primary care outpatients Spain	South/Central America	- PRIME-MD	Somatoform disorder prevalence: Latin-American migrants (18.1%); native Spanish (6.6%). No significant group differences when adjusting for sociodemographic and social support variables.
Schweitzer et al., 2011	To investigate the contributions of pre-migration and post-migration factors in predicting mental health among Burmese refugees.	*N* = 70 ♀ ♂ Age = 18–80 Burmese refugee Australia	Myanmar	- HSCL-37*	Somatization prevalence: 37%. Pre-migration trauma events (*p* < 0.05), traumatization (*p* < 0.01), and PMLD (*p* < 0.01) were found correlated with somatization.
Shiroma and Alarcon, 2011	To examine the possible connection between demographic factors and acculturation level with somatization.	*N* = 180 ♀ ♂ Age = 20–60 Chronically mentally ill immigrants US	Latin America, Russia	- SCL-90-R*	Higher somatization: Russians > Hispanics; high school or above education; lower acculturation; shorter length of residence in the US (only among Russians).
Small et al., 2003	To explore cultural assumptions about somatization in three groups of immigrant women who had recently given birth in Melbourne.	*N* = 318 ♀ Age = n/a Women who had recently given birth. Australia	Vietnam, Turkey, Filipino	- SF-36*	Levels of somatic symptoms: Turkish > Vietnamese, Filipino.
Spiller et al., 2016	To examine factors associated with increased symptom severity of PTSD.	*N* = 134 ♀ ♂ Age >18 Psychiatric outpatients Switzerland	Various ethnic groups	- SCL-90-R*	Somatization + PTSD: positive association (*p* < 0.01). PTSD symptoms were mainly predicted by somatization (*p* < 0.001).
Stewart et al., 2012	To evaluate if migrant women who experienced violence associated with pregnancy had a difference health profile compared to other childbearing migrant women.	*N* = 774 ♀ Age (M) = 28.3 Pregnant migrants Canada	South/Central America, Africa, Europe	- HSCL-37*	Somatization prevalence: abused migrants > non-abused migrants. Somatization + social support: no significant association.
Van Wyk et al., 2012	To examine the impact of therapeutic interventions for people from refugee backgrounds within a naturalistic setting.	*N* = 62 ♀ ♂ Age = 18–80 Adult refugees Australia	Myanmar	- HSCL-37*	Therapeutic intervention improved somatization (Effect size *r* = 0.60).
Whitley et al., 2006	To explore illness narratives, explanatory models, symptom-attribution and help-seeking in the community.	*N* = 15 ♀ ♂ Age: 21–67 Community sample Canada	West Indies	- MINI	MUS were ascribed to the chronic effect of overwork, lack of routine and irregular patterns of daily living.

BSI, Bradford Somatic Inventory; DSM-IV, Diagnostic and Statistical Manual of Mental Disorders 4th ed.; SCID-RV, Structured Clinical Interview for DSM-IV Axis I Disorders – Research Version; MSPQ, Modified Somatic Perception Questionnaire; SCL-90-R, Symptom Check List-90 Items-Revised; HSCL-37, Hopkins Symptom Checklist-37; DIS, Diagnostic Interview Schedule Somatization Disorder Module; SOMS-II, Screening for Somatoform Symptoms-II; SSI, Somatic Symptoms Index; PHQ-15, Patient Health Questionnaire; PRIME-MD, Primary Care Evaluation of Mental Disorders; BSI, Brief Symptom Inventory; SF-36, SF-36 Health Survey; MINI, McGill Illness Narrative Interview. ^*^Authors specified in which language the questionnaires were administered and whether the scales were adapted to the language of participants.Authors specified in which language the questionnaires were administered and whether the scales were adapted to the language of 836 participants.

## Results

**Figure 1:**
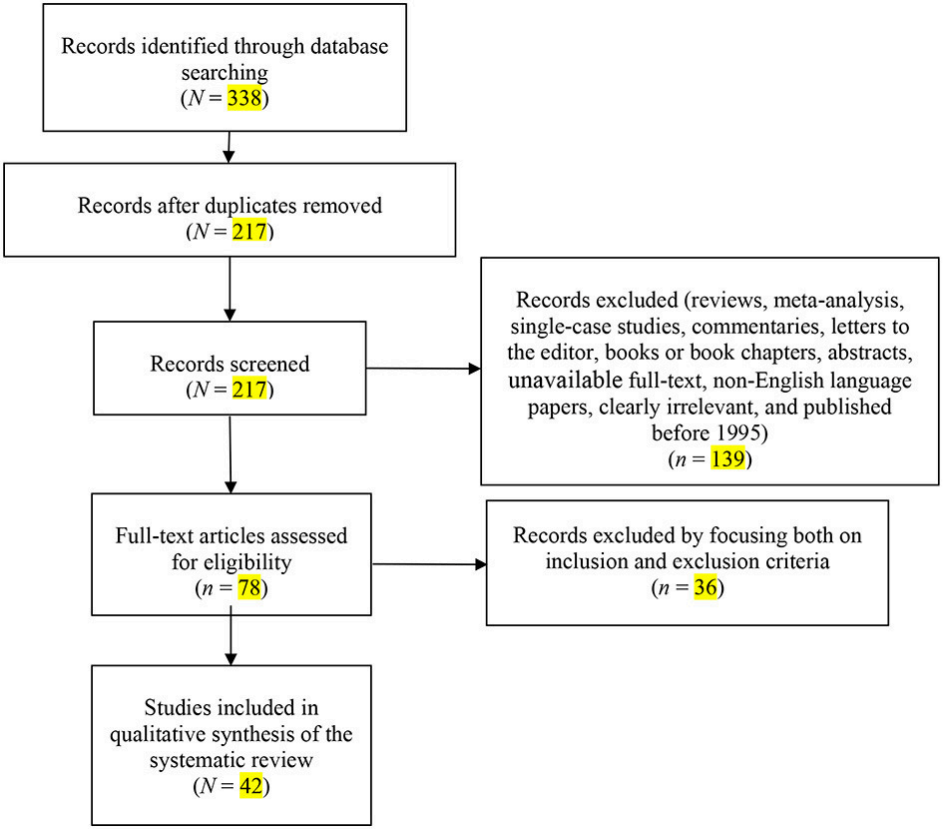
PRISMA Flowchart of the systematic search.

The search of electronic databases initially yielded *N* = 338 citations, as reported in the PRISMA flowchart (Figure 1). After removing the duplicates, *N* = 217 records remained. Of these, *n* = 139 citations were eliminated as they were reviews, meta-analyses, commentaries, letters to the editor, books or book chapters, abstracts, or non-English language papers, or because they did not meet the inclusion criteria. Of the 78 full text articles assessed for eligibility, *n* = 36 studies were excluded by focusing both on inclusion and exclusion criteria. Ultimately, *N* = 42 studies were selected for inclusion in the systematic review (see Table 1 for a detailed description of the reviewed studies).

The reviewed studies were published between 2000 and 2017. These 42 papers reported the results of 38 cross-sectional analyses and four longitudinal analyses. In this section, the studies are mainly grouped and described based on the characteristics of migrant populations at risk for somatization.

### Measurement and Assessment of Somatization

Table 1 summarizes the measurement and assessment of somatization in the included studies. Twenty-nine records used self-report questionnaires; of these, 22 specified in which language the questionnaires were administered and whether the scales were adapted to the language of the participants (see Table 1). Thirteen articles (Mak and Zane, 2004; Nickel et al., 2006; Cwikel et al., 2008; Sachs et al., 2008; Shiroma and Alarcon, 2011; David et al., 2012; Heredia Montesinos et al., 2012; Mölsä et al., 2014, 2017; Rask et al., 2015, 2016; Spiller et al., 2016; Choi et al., 2017) used the Symptom Checklist-90-R (SCL-90-R) Somatization subscale (Derogatis, 1977). The SCL-90-R consists of nine subscales aimed at measuring psychopathology, including somatization. Seven studies (Aragona et al., 2005, 2008, 2010, 2011, 2012, 2013; Deisenhammer et al., 2012) assessed somatization using the Bradford Somatic Inventory (BSI-21) (Mumford et al., 1991), a widely validated self-assessment questionnaire specifically designed for transcultural research and formerly used to assess somatization among groups of primary care immigrants. The Patient Health Questionnaire-15 (PHQ-15) (Dreher et al., 2017; Mendoza et al., 2017; Morawa et al., 2017) and the Hopkins Symptom Checklist-37 (HSCL-37) (Breuer and Freud, 1893–1895; Alexander, 1950; Derogatis, 1977; Mai and Merkey, 1980; Barsky and Klerman, 1983; Bridges and Goldberg, 1987; Lipowski, 1988; Escobar et al., 1989; Mumford et al., 1991; American Psychiatric Association, 1994, 2013; Castillo et al., 1995; Escobar, 1995; Gureje et al., 1997; Kirmayer and Young, 1998; Silove et al., 1998; Simon et al., 1999; Mak and Zane, 2004; Aragona et al., 2005, 2008, 2010, 2011, 2012, 2013; Nickel et al., 2006; Kirmayer and Sartorius, 2007; Cwikel et al., 2008; Jackson and Kroenke, 2008; Sachs et al., 2008; Liberati et al., 2009; Bermejo et al., 2010; Beirens and Fontaine, 2011; Perruchoud and Redpath-Cross, 2011; Schweitzer et al., 2011; Shiroma and Alarcon, 2011; David et al., 2012; Deisenhammer et al., 2012; Heredia Montesinos et al., 2012; Stewart et al., 2012; Van Wyk et al., 2012; Bragazzi et al., 2014; Mölsä et al., 2014, 2017; Rohlof et al., 2014; Haller et al., 2015; Rask et al., 2015, 2016; Spiller et al., 2016; Choi et al., 2017; Dreher et al., 2017; Mendoza et al., 2017; Morawa et al., 2017; Radl-Karimi et al., 2018) were each used in three records. The PHQ-15 consists of four subscales: Somatoform Disorder, Depressive Disorder, Panic Disorder, and Functioning of the Patient. The HSCL-37 measures symptoms along three subscales: Anxiety, Depression, and Somatization (Derogatis et al., 1974). The other questionnaires, i.e., Screening for Somatoform Symptoms-II (SOMS-II) (Heredia Montesinos et al., 2012), the Somatic Symptoms Index (SSI) (Mak and Zane, 2004), the SF-36 Health Survey (SF-36) (Small et al., 2003), the Modified Somatic Perception Questionnaire (MSPQ) (Bragazzi et al., 2014), and the Brief Symptom Inventory (BSI) somatization subscale (Ritsner et al., 2000) were used in one article each. The SOMS-II evaluates somatization disorder according to DSM-IV (Rief et al., 1997). The SSI measures somatization disorder and is based on the DSM classification (Escobar et al., 1989). The SF-36 provides a measure of physical, mental, and social functioning (Ware and Sherbourne, 1992). The MSPQ is a scale used to measure somatization and to investigate body perception and physiologic functions (Main, 1983). The BSI was developed from the SCL-90-R; its Somatization subscale measures the distress arising from perceptions of bodily dysfunction (Derogatis and Spencer, 1982). Four studies (Bäärnhielm and Ekblad, 2000; Miranda et al., 2005; Fenta et al., 2006; Salinero-Fort et al., 2015) assessed somatization administering standardized semi-structured interviews based on the DSM-IV classification, i.e., Structured Clinical Interview for DSM-IV Axis I Disorders – Research Version (SCID-RV) (Bäärnhielm and Ekblad, 2000), Diagnostic Interview Schedule (DIS) Somatization Disorder Module (Fenta et al., 2006), and Primary Care Evaluation of Mental Disorders (PRIME-MD) (Miranda et al., 2005; Salinero-Fort et al., 2015). Each aims to give a measure of common mental health problems, including somatoform disorders (Swartz et al., 1986; Spitzer et al., 1994; First et al., 1997). Whitley et al. (Whitley et al., 2006) administered the McGill Illness Narrative Interview (MINI) (Groleau and Kirmayer, 2004). The MINI has been used in cross-cultural research in many cultures and contexts to explore diverse health issues and conditions, including MUS. Six records assessed somatization using unstandardized semi-structured interviews (Hondius et al., 2000; Mirdal, 2006; Perron and Hudelson, 2006; Karasz et al., 2007; Beirens and Fontaine, 2011; Borra, 2011) mainly focused on: MUS (Perron and Hudelson, 2006; Karasz et al., 2007), emotional distress associated with physical symptoms (Beirens and Fontaine, 2011), and somatic complaints (Hondius et al., 2000; Mirdal, 2006; Borra, 2011). Finally, in two studies conducted by Nadeem et al. (Nadeem et al., 2008, 2009), participants were classified as somatizers when they reported six or more DSM-IV symptoms of somatization disorder.

### International Migrants Compared to Host Country Natives

Eight studies did not find significant differences in levels of somatization between migrant and local populations. In a study undertaken to assess the prevalence of mental disorders in 1,594 outpatients seen in primary care, Salinero-Fort et al. (2015) found that the prevalence of somatoform disorders was significantly higher (*p* < 0.001) among Latin American immigrants (18.1%) than in Spanish native-born outpatients (6.6%); however, the association became not statistically significant after adjusting for sociodemographic variables. Miranda et al. (2005) compared 9,151 low-income African-born, Caribbean-born, and US native-born black women on rates of somatic symptoms. Rates of somatization were similar across the three groups (3.2, 3.3, and 2.8%, respectively). In two studies, Nadeem et al. (2008, 2009) recruited low-income immigrant and US-born women with perceived mental health problems. In both studies the groups did not differ significantly in somatization rates. Two studies conducted in Finland (Mölsä et al., 2014, 2017) investigated the presence of somatization in older Somali refugees and pair-matched Finnish controls; no group differences were found. In a comparative study, David et al. (2012) investigated group differences in somatization using a sample of 753 immigrant and German native-born women treated for hyperemesis gravidarum. Although the number of immigrant women treated for hyperemesis gravidarum was higher compared to the resident population, both groups showed high levels of somatization. Finally, in a study that compared 576 immigrants of different ethnic groups and 400 Israeli native-born patients accessing primary care clinics (Mirdal, 2006), no significant group differences in rates of somatization were found.

Somatization was found to be significantly prevalent in immigrants in only four studies that compared samples of immigrants with the host country's natives (Beirens and Fontaine, 2011; Deisenhammer et al., 2012; Bragazzi et al., 2014; Dreher et al., 2017). An Italian study (Bragazzi et al., 2014) investigated the differences in somatic perception between a group of 143 immigrant outpatients from South America and Africa vs. a control group of 186 Italian outpatients. After adjusting for gender and age differences, the immigrant group showed significantly higher mean scores of somatic disturbances than the control group (*p* < 0.01). In a comparative study involving Vietnamese immigrant and native German outpatients (Dreher et al., 2017), 32% of Vietnamese patients were classified as suffering from severe somatic symptoms, while only 12.8% of the German patients reported severe somatic symptoms (*p* < 0.001). Deisenhammer et al. (Deisenhammer et al., 2012) compared 40 Turkish immigrant women, 55 Turkish women residing in Turkey, and 41 Austrian native-born women. Results showed that Turkish immigrants had the highest prevalence of somatic symptoms, though not significantly higher than Turkish persons living in Turkey. Beirens and Fontaine (2011), who investigated somatization-related complaint differences between 144 Turkish immigrants, 222 Turkish living in Turkey, and 353 Belgians native-born, found that Turkish groups living in Turkey reported a higher tendency to somatize, followed by Turkish immigrants and Belgians.

### Sociodemographic and Cultural Predictors of Somatization

Thirteen studies investigated the relationship between specific sociodemographic characteristics and somatization in immigrants. Reports showed that being female (Ritsner et al., 2000; Mak and Zane, 2004; Aragona et al., 2005, 2008, 2012; Fenta et al., 2006; Cwikel et al., 2008; Bragazzi et al., 2014; Rask et al., 2016; Morawa et al., 2017), older (Ritsner et al., 2000; Mak and Zane, 2004; Mölsä et al., 2014, 2017), and having low language proficiency (Dreher et al., 2017; Morawa et al., 2017) are significant and common sociodemographic risk factors for somatization among immigrants.

Conflicting results have been found on marital status (Ritsner et al., 2000; Aragona et al., 2008), education level (Aragona et al., 2008; Shiroma and Alarcon, 2011), acculturation level (Mak and Zane, 2004; Shiroma and Alarcon, 2011), length of residence (Ritsner et al., 2000; Mak and Zane, 2004; Shiroma and Alarcon, 2011; Aragona et al., 2012), and economic satisfaction (Mirdal, 2006; Choi et al., 2017), relative to somatization. The effect of education level on somatization was investigated in an American study involving 1,747 Chinese immigrants (Mak and Zane, 2004). The authors found that the experience of somatization was more prevalent among individuals with less than college education. In contrast, in a study involving 180 Russian and Latino immigrants in the US (Shiroma and Alarcon, 2011), high school or above levels of education were found to be significantly associated with higher somatization. The effect of marital status on somatization was investigated in an Italian study involving 301 outpatients of various ethnic groups (Aragona et al., 2008). The authors showed a significantly increased risk for somatization among Caucasian-married subjects (*p* = 0.035). Ritsner et al. (2000) examined somatic distress and its correlation with specific demographic characteristics in 966 Russian-born Jews who had migrated to Israel. Overall, the prevalence of somatization was 21.9%; divorced and widowed respondents, compared to married and single respondents, were more likely to meet the criteria for somatization. The authors also found that longer length of residence in the host country was associated with higher levels of somatization symptoms (*p* < 0.0001). A cross-sectional study (Shiroma and Alarcon, 2011), involving Russian and Hispanic immigrants living in the US, found conflicting results. In the univariate and multivariate analyses, shorter length of stay in the US was significantly related to somatization (though only among Russians; *p* < 0.001) and higher somatization scores were significantly related to lower acculturation (*p* < 0.001) within both groups. Conversely, in a study of 1,747 Chinese Americans in Los Angeles County, length of residence in the host country and acculuration were not related to somatization (Mak and Zane, 2004). In this investigation, the prevalence of somatization for the total sample was 12.9%. Aragona et al. (2012) assessed differences in somatization among Europeans, Asians, South Americans, and Africans living in poor social conditions. Among the 3,051 recruited outpatients, 25.6% were somatizers, but there were no significant differences in the duration of permanence in Italy and immigrant regular/irregular status. Focusing on economic satisfaction, a follow-up study conducted in the US (Mirdal, 2006) showed that, although living conditions of subjects had improved (i.e., economic independence and social improvement) during the last 20 years, and the number of somatic complaints had decreased, levels of distress were still high. In a study examining the relationship between somatization and life satisfaction of North Korean refugees resettled in South Korea (Choi et al., 2017) somatization was found to be related only to the economic satisfaction domain (*r* = −0.19, *p* < 0.01).

Six of the examined studies compared immigrants of different ethnic groups and considered the associations between ethnicity or other sociodemographic factors and somatization.(Aragona et al., 2005) evaluated the prevalence of somatization in a sample of 301 immigrants of four ethnic groups (Caucasian, Asian, South-Central American, and African) attending a primary care service in Italy. The prevalence of somatization in the total sample was found to be 35.2%. Somatization was significantly higher in South-Central Americans than in other ethnic groups (*p* = 0.012). In a subsequent study involving the same sample (Aragona et al., 2008), subgroup analysis of the ethnic groups showed a significantly increased risk for somatization only for Caucasian (*p* = 0.001) and South-Central American (*p* = 0.003) women and Caucasian married persons (*p* = 0.035). In another multicultural study conducted in Italy, Aragona et al. (2012) assessed the differences in somatization among immigrant outpatients living in poor social conditions. Among the 3,051 recruited participants, 25.6% were somatizers, the greatest proportion of whom were from South America (30.1%), followed by Europeans (23.2%), Africans (21.2%), and Asians (16.3%). Specifically, the authors found that somatization occurred more frequently in Peruvians (32.9%). Small et al. (2003) compared 107 Turkish, 104 Vietnamese, and 107 Filipino women who had recently given birth in Australia. Results showed that Turkish women were the most likely of the three groups to report high levels of somatic symptoms, followed by Vietnamese and Filipino women. In two studies, Rask et al. (2015, 2016) assessed and compared the prevalence of mental health symptoms among Russian, Somali, and Kurdish immigrants in Finland. The prevalence of somatization was 14.8% for Russians, 12.9% for Somalians, and 28.9% for Kurds (Rask et al., 2016). The authors also reported that somatization increased the odds for mobility limitation within all migrant groups (Russians OR 4.29; Somalis OR 18.83; Kurds OR 3.53) (Rask et al., 2015).

### Clinical Psychological Features of Immigrants at Somatization Risk

To date, ten investigations have examined the association between somatization, general psychological distress, and other vulnerability or protective psychological factors in individuals with migratory backgrounds. Three studies found positive associations between psychological distress and a wide range of somatic complaints in depressed patients (Mak and Zane, 2004; Borra, 2011; Heredia Montesinos et al., 2012). Borra (2011) showed higher levels of psychological distress in Turkish depressed women living in the Netherlands who reported somatic symptoms than Turkish depressed women without somatic symptoms. Heredia Montesinos et al. (2012) showed significant correlations between depression (*p* < 0.101), overall psychological distress (*p* < 0.001), and somatic symptoms in Turkish depressed women living in Germany. Mak and Zane (2004) found similar results in a sample of 333 Turkish immigrants in Germany, where 24.2% of the total sample exhibited severe levels of somatization. Among these somatizing persons, 53.1% also reported comorbid severe levels of depression (*r* = 0.74).

In a Swedish qualitative study, Bäärnhielm and Ekblad (2000) found that Turkish migrant participants (*N* = 10) experienced and communicated psychological distress in the form of physical symptoms, even when somatic diagnoses were present. Distress was communicated by concrete expressions about the body, emotions, and social and life situations. The participants' illness attribution patterns were mostly characterized by not verbalizing causal explanations, but rather links of coherence between health and various aspects of life. Ritsner et al. (2000) investigated the relationship between psychological distress and somatization in an immigrant population in Israel. The co-occurrence of these factors was 20.4%. Somatization was positively correlated with the intensity of psychological distress (*p* < 0.001). Similar results were found in a study involving a representative community sample of Chinese Americans (Mak and Zane, 2004); it was reported that anxiety (*p* < 0.001), depression (*p* < 0.001), adverse lifetime events (*p* < 0.05), and social support (*p* < 0.05) were significantly related to somatization.

Focusing on perceived social support, in a recent study (Mendoza et al., 2017) that evaluated the role of migration stressors on poor mental health among Filipino female domestic workers in China (*N* = 261), Mendoza et al. (2017) applied hierarchical multiple regression analysis to test for direct and moderating effects of social networks on psychological distress. Post-migration stress was significantly and positively correlated with somatization symptoms (*p* < 0.001) and with somatization symptom severity (*p* < 0.01). Social network support from family was not associated with somatization, nor did it modify the association between stress and these symptoms. Social network support from friends, however, was positively associated with somatization and significantly moderated the relationship between stress and these symptoms (*p* < 0.01). Stewart et al. (2012), investigating a sample of pregnant immigrant women, found that although abused women were more likely to have inadequate social support and to report more depression, anxiety, somatization, and PTSD (*p* < 0.001), social support status did not affect somatization.

Two studies examined the effect of cultural differences on somatization in immigrants, taking into account specific clinical-psychological features. A study that compared Turkish persons living in Belgium, Turkish living in Turkey, and Belgians native-born. Turkish majorities scored higher on all somatic factors, anxiety-sadness, and self-conscious emotions followed by Turkish immigrants and Belgian majorities (Beirens and Fontaine, 2011). Indeed, the authors found a mediation effect of anxiety-sadness and self-conscious factors on the differences in somatic factors only between Belgians and non-migrated Turkish persons (Beirens and Fontaine, 2011). Sachs et al. (2008) explored the experiences, coping strategies, and psychological distress of Tibetan refugees in India who reported trauma exposure. The authors used data on coping strategies and cognitive appraisal of experience severity to test the hypothesis that these mechanisms mediate psychological outcomes. Participants reported notably low psychological and somatic symptoms; thus, coping activity (primarily religious) and subjective appraisals of the severity of their experiences (i.e., social comparison) appeared to mitigate the psychological effects of trauma exposure.

Finally, only two studies sought to evaluate the effects of treatment interventions on somatization in immigrants. In a 6-week randomized prospective controlled trial aimed at examining whether bioenergetic exercises significantly influenced the inpatient psychotherapeutic treatment results for 128 Turkish immigrants with chronic somatoform disorders, this activity appeared to improve symptoms of somatization (Nickel et al., 2006). A longitudinal non-randomized study (Van Wyk et al., 2012) examined the impact of therapeutic interventions of mental health conducted with the aim of facilitating adjustment and acculturation for adult Burmese refugees within a naturalistic setting in Australia. Over the course of the interventions, participants experienced a significant decrease in symptoms of PTSD, anxiety, depression, and somatization (*r* = 0.60).

### Somatization and Health Behavior

Six studies explored the associations between somatization and variations in perceptions of health, service utilization patterns, and treatment preferences in migrant populations. Karasz et al. (2007) investigated cultural differences in illness experience using a sample of immigrants divided into two groups: European Americans (*n* = 36) and South Asians (*n* = 35). The groups reported similar symptoms, but the organization of illness episodes and explanatory models associated with these episodes differed sharply. Twenty percent of all European American psychological illness problems (but none of South Asian problems) were explained entirely by physical or somatic causes. Moreover, 43% of European American psychological problems included at least one physical cause, while only 4% of psychological problems in the South Asian group included at least one physical cause.

The health service utilization patterns of immigrants and refugees were analyzed by Fenta et al. (2006) in a sample of 342 Ethiopians residing in Canada. The authors found that 63.2% of the respondents had experienced one or more somatic symptom(s) in the previous 12 months. The number of somatic symptoms experienced was positively associated with increased rate of medical services utilization (*p* < 0.05) and with increased utilization of nonmedical services (e.g., religious leaders, traditional healers, and other non-health professionals; *p* < 0.001). Ritsner et al. (2000) investigated the relationship between psychological distress and somatization symptoms and healthcare-seeking behavior. Somatization was positively correlated with self-reported poor health and with healthcare-seeking behavior (*p* < 0.001). Additionally, Mak and Zane (2004) found that a significantly higher percentage of Chinese American somatizers rated their health as poor or fair, compared to non-somatizers (*p* < 0.0001) and reported seeking help from both traditional Chinese and Western medicine (*p* < 0.01). In two studies, Nadeem et al. (2008, 2009) compared low-income immigrants with US-born women with acknowledged mental health problems to investigate the differences in treatment preferences and perceived need for care. In the first study, somatization was found to be positively associated with endorsing medication (*p* < 0.05) and faith (*p* < 0.05) as a helpful treatment, with no significant differences between ethnic groups (Nadeem et al., 2008). The subsequent study (Nadeem et al., 2009) involved 1,577 low-income immigrant and US-born women with depression and found that having multiple somatic symptoms increased the likelihood of endorsing perceived need for care compared with having few somatic symptoms (*p* < 0.001), across all the ethnical groups.

### The Clinical Link Between Trauma and Somatization

Immigrants frequently experience multiple traumatic events in pre-migration as well as post-migration life. Hondius et al. (2000), in a study aimed at estimating the contribution of different forms of violence to the health complaints of refugees, confirmed that high frequencies of torture events and substantial numbers of medical complaints were common among immigrants. Specifically, the authors found that refugees attributed their somatic and psychological complaints to torture (29%) and to worries related to PMLD (40%). A positive correlation between somatization and the number of previous traumatic events (*r* = 0.33, *p* < 0.001) was also observed in North Korean refugees resettled in South Korea (Choi et al., 2017). Similarly, studies that compared mental and somatic health among 256 elderly Somali refugees and Finnish controls found that high levels of pre-migration traumatic events were associated with high levels of somatization symptoms (*p* < 0.01) (Mölsä et al., 2014, 2017).

Several studies have shown significant associations between somatization, traumatic events, PTSD and PMLD. Pre-migration traumatic experience and PTSD are both frequently observed in immigrant somatizers. Many studies (Aragona et al., 2010, 2011, 2013; Schweitzer et al., 2011; Mölsä et al., 2014) have found a comorbidity between PTSD and somatization, ranging from 30.7% to 80%. Aragona et al. (2010, 2011, 2013) identified a high prevalence of somatization in a sample of immigrants who had experienced traumatic events, in three studies undertaken in 2010, 2011, and 2013. The first study (Aragona et al., 2010) conducted on 101 immigrant outpatients attending a primary care service, found that the number of somatizers reporting at least one traumatic event (69.2%) was significantly higher than that of non-somatizers (40.3%). In the second study (Aragona et al., 2011), conducted on the same sample, the authors found that having PTSD was significantly more common in somatizers (30.7%) than in non-somatizers (6.4%). This study also reported that the number of somatizers having serious or very serious PMLD was significantly higher than that of non-somatizers (*p* = 0.016). Finally, the third study (Aragona et al., 2013), conducted on 391 immigrant outpatients, found that patients with PTSD had highest potentially traumatic events rates (49.95%), PMLD rates (56.94%), and somatization rates (80%). Consistent with these findings, Schweitzer et al. (2011) investigated the contributions of pre-migration and post-migration factors in predicting mental health among Burmese refugees in Australia. In this study, a substantial proportion of participants reported PTSD (9%) and somatization (37%). Pre-migration trauma events (*p* < 0.05), traumatization (*p* < 0.01), and PMLD (*p* < 0.01) were found to be correlated with somatization. Stewart et al. (2012) recruited 774 pregnant immigrant women to evaluate whether immigrant women who experienced violence associated with pregnancy had a different health profile compared to other childbearing immigrant women. The study showed that immigrant women who reported abuse associated with pregnancy (7.6%) were more likely to have symptoms of somatization (*p* < 0.001) and PTSD (*p* < 0.001).

A positive correlation between somatization and the severity of PTSD symptoms is also reported. Spiller et al. (2016) conducted a cross-sectional study to examine the factors associated with increased symptom severity of PTSD in 134 severely traumatized refugees. Somatization was found to be significantly related to PTSD (*p* < 0.01), trauma exposure (*p* < 0.01), and PMLD (*p* < 0.01). Specifically, PTSD symptoms were mainly predicted by somatization (*p* < 0.001) and anger (*p* < 0.001). Interestingly, only a cross-sectional study (Sachs et al., 2008) involving 769 Tibetan refugees found that levels of somatization were extremely low, despite the high prevalence of potentially traumatizing events. The authors observed that coping activity appeared to mediate the effects of trauma exposure on psychological distress [*F*_(2, 763)_ = 17.96, *p* < 0.001, *R*^2^ = 0.02].

Finally, two studies focused on the illness narratives of immigrants suffering from somatic, emotional or MUS with the aim of exploring how immigrants made sense of their suffering. A Canadian study (Whitley et al., 2006) highlighted that West Indian immigrants ascribed their MUS almost exclusively to the chronic effect of post-migratory factors (overwork, lack of routin,e and irregular patterns of daily living). By contrast, a Swiss study (Perron and Hudelson, 2006) showed that Yugoslav asylum seekers attributed the onset of somatic symptoms to past traumatic experiences such as war, flight, and loss of loved ones, and talked about current difficult life conditions (financial worries, concerns about their children, uncertainty about the future, fear of expulsion, and lack of social support) as perpetuating their symptoms and posing barriers to improvement.

## Discussion

The present study aimed to systematically investigate published original research reports, evaluating the emerging clinical links between migration and somatization by providing a qualitative data synthesis of the studies. The main findings of this study are that migrants with somatization were more psychologically distressed, had an increased perceived need for healthcare service utilization, and reported more PMLD and/or PTSD than those without somatization. Specific individual features mediated the association between somatization and migration. The prevalence and correlates of somatization were found to vary across the immigrant groups, depending on cultural variation, in reasons for migration, stress exposure, explanatory models of illness, coping, and other individual variables.

In our first hypothesis, somatization would be significantly associated with migration because of the supposed high exposure to stressful experiences in individuals with migratory backgrounds. Rates of somatization in immigrants ranged between 12.9 and 67% (Nadeem et al., 2009; Rask et al., 2016). As shown in the results reported in the collected articles, there was an extreme variability in the association between somatization and migration according to the heterogeneity of the studied migrant populations, both in terms of mental health as well as other individual variables. Being female, older, and having low language proficiency are significant sociodemographic risk factors for somatization among immigrants (Ritsner et al., 2000; Mak and Zane, 2004; Aragona et al., 2005; Bragazzi et al., 2014; Dreher et al., 2017; Morawa et al., 2017). This suggests that some sociodemographic variables may represent specific risk factors for somatization across all ethnic groups. Studies showed conflicting results when taking into account other sociodemographic variables such as length of residence (Ritsner et al., 2000; Mak and Zane, 2004; Shiroma and Alarcon, 2011; Aragona et al., 2012), income (Mirdal, 2006; Choi et al., 2017), acculturation (Mak and Zane, 2004; Shiroma and Alarcon, 2011), education level (Mak and Zane, 2004; Shiroma and Alarcon, 2011), and marital status (Ritsner et al., 2000; Aragona et al., 2008). Additionally, some studies (Miranda et al., 2005; Mirdal, 2006; Nadeem et al., 2008, 2009; David et al., 2012; Mölsä et al., 2014, 2017) did not find significant differences in levels of somatization between migrants and the host country's natives; alternatively, the differences became not statistically significant after adjustment for sociodemographic confounding variables (Salinero-Fort et al., 2015). These findings could be explained by the “healthy migrant” effect (Razum et al., 2000). Several studies have suggested that recent immigrants are generally healthier than native-born populations, notwithstanding that they frequently have a lower socioeconomic status and less access to health care services. This “epidemiological paradox” is usually attributed to a self-selection process prior to migration, “cultural buffering,” and official health screening and employability in receiving countries (Domnich et al., 2012). Another consideration is that the somatization disparities became not statistically significant when migrant and native populations were recruited from psychiatric (Nadeem et al., 2008, 2009), socioeconomic (Miranda et al., 2005), and clinically disadvantaged settings (Mirdal, 2006; David et al., 2012).

By contrast, somatization was found to be significantly prevalent in immigrants in only four studies that compared samples of immigrants with the host country's natives (Beirens and Fontaine, 2011; Deisenhammer et al., 2012; Bragazzi et al., 2014; Dreher et al., 2017). Among these, only two studies (Beirens and Fontaine, 2011; Deisenhammer et al., 2012) tried to clarify the independent relationship between migration and somatization, by comparing individuals of the same nationality with and without migratory backgrounds. Contrary to expectations, results showed that levels of somatization in migrants were not significantly higher than those reported by non-migrant individuals with the same nationality. These results could be explained by the “health selection hypothesis.” This construct suggests that immigrants tend to be different from their compatriots who do not migrate (Chiquiar and Hanson, 2002; Chiswick et al., 2008). Thus, immigrants may be more educated, less risk exposed, more entrepreneurial and better prepared to confront stressful situations (Anderson et al., 2004). Instead, some studies (Small et al., 2003; Karasz et al., 2007; Aragona et al., 2008, 2011; Sachs et al., 2008; Schweitzer et al., 2011; Shiroma and Alarcon, 2011; Deisenhammer et al., 2012; Rask et al., 2015, 2016) have underscored that the impact of life events, sociodemographic and clinical features, and the prevalence of somatization and its symptomatology varied between different ethnic groups. For example, in three multicultural studies (Aragona et al., 2005, 2008, 2012), the likelihood of somatization varied widely among the different groups and was significantly higher in Latin Americans. These results suggest that the relationship between somatization and migration is particularly complex and culturally mediated; hence, any diagnosis or treatment of the individual with migratory background must be grounded in some knowledge of the person's ethnic origin. Postulating the existence of such an intimate and harmonious connection between somatization and ethnicity, however, overlooks a pivotal distinction: while it is true that ethnic variations can and do affect psychopathological presentations, some pathogenic features are so overwhelming that they will be expressed in any environment.

In our second hypothesis, the prevalence and correlates of somatization would be different, based on cultural variation in reasons for migration, trauma exposure, coping, and explanatory models of illness across immigrant groups and receiving contexts. Most of the examined papers (Hondius et al., 2000; Sachs et al., 2008; Aragona et al., 2010, 2011, 2013; Stewart et al., 2012; Choi et al., 2017; Mölsä et al., 2017) reported that immigrants use somatization to express their distress associated with pre-migration (e.g., material deprivation, religious persecution, torture, sexual abuse, being forced to harm others, loss of loved ones) and to post-migration (e.g., difficulties in accessing health and welfare services, difficulties in finding work or bad job conditions, stressors linked to the acculturation process, poverty, and discrimination) adverse life events. Pre-migratory traumatic events may have ongoing indirect effects by increasing the vulnerability of immigrants to future stressors, thus leading to more frequent PMLD. The presence and amount of PMLD are positively correlated with somatization (Aragona et al., 2011; Schweitzer et al., 2011). Moreover, PMLD in somatizers may exacerbate an existing predisposition to PTSD caused by exposure to pre-migration trauma (Schweitzer et al., 2011). This relation between pre-migratory traumas, PTSD, PMLD, and overall psychopathological symptoms is relevant because it stresses that traumatic experiences are key factors in immigrant psychopathology. Patients attribute their symptoms to past traumatic experiences and believe that PMLD contributes to their chronicity (Perron and Hudelson, 2006; Whitley et al., 2006). As a result, they formulate their suffering in both medical and social or legal terms, seeking help from physicians for all of them (Perron and Hudelson, 2006). Somatization increases the perceived need for care and health care service utilization (Nadeem et al., 2009). Moreover, increased rates of medical service utilization (especially family doctors) and increased utilization of non-medical services (i.e., traditional healers, religious leaders) were found to be strongly associated with somatization in immigrants (Ritsner et al., 2000; Fenta et al., 2006; Nadeem et al., 2008). Although somatizing immigrants tend to present high levels of help-seeking behaviors (Ritsner et al., 2000) and social interactions generally appear to play an important role in mental health and wellness for immigrants (Ahn et al., 2017), studies have shown that perceived social support did not affect somatization when post-migration stress (Mendoza et al., 2017) or traumatic experiences (Stewart et al., 2012) occur.

Studies have suggested that immigrants all over the world experience significantly more stressful life events, negative emotions, and psychological distress than non-immigrants, and therefore have a higher risk of somatization (Buchwald et al., 1986; Castillo et al., 1995). The tendency to somatize emotional distress was associated with poor mental health and quality of life in migrant populations (Mirdal, 2006; Rask et al., 2015, 2016; Choi et al., 2017). Studies have also found a comorbidity between severe depression and somatization (Mak and Zane, 2004; Borra, 2011; Heredia Montesinos et al., 2012; Morawa et al., 2017). Moreover, research has shown that the tendency to report physical complaints could be an expression of overall psychological distress and depressive symptoms in immigrants (Borra, 2011; Deisenhammer et al., 2012; Heredia Montesinos et al., 2012).

However, negative emotions seem to be associated with somatization, independent of the migration factor (Beirens and Fontaine, 2011). The explanatory models of illness episodes may differ sharply among different cultural groups, yet psychological attribution is rarely accepted; instead, individuals tend to communicate distress through concrete expressions about the body (Bäärnhielm and Ekblad, 2000; Karasz et al., 2007). From this perspective, somatization may not necessarily be a pathological mechanism among migrant populations, but rather a product of cultural differences.

Caution, however, should be exercised when interpreting the findings of this systematic review because of the limits of the reviewed studies. Overall, studies prevalently adopted a cross-sectional design (*n* = 38), used only one method for assessing somatization (Ritsner et al., 2000; Small et al., 2003; Mak and Zane, 2004; Heredia Montesinos et al., 2012; Bragazzi et al., 2014), and are difficult to compare because different definitions for somatization were applied. Different somatization measures were used, with different cutoff points for somatization. In addition, most studies did not look at coexisting somatic disorders; a thorough somatic examination was rarely included. Thus, in most cases, a full diagnosis of the somatic symptom disorder could not be reached. Moreover, 29 studies used self-report questionnaires to evaluate somatization; among these, seven (Mak and Zane, 2004; Cwikel et al., 2008; Heredia Montesinos et al., 2012; Mölsä et al., 2014, 2017; Rask et al., 2015, 2016) did not specify in which language the scales were administered or whether the scales were adapted to the language of the participants. Therefore, it would be advisable for future studies to use the same instruments, with consistent cutoff points for somatization. When translated, there should be a back-translation, and after that a validation of the questionnaire.

During our examination of the environmental factors related to health in immigrants, it became clear that there was a lack of tailored therapies that included psychological, social, and legal assistance with the aim of promoting adjustment and acculturation, improving mental health, and mitigating the symptoms of somatization in immigrant patients. Indeed, only two studies sought to evaluate the effects of treatment interventions on somatization in immigrants (Nickel et al., 2006; Van Wyk et al., 2012). In addition, as patients and health care professionals face differences in cultural backgrounds (e.g., linguistic barriers, variant health/illness beliefs, different medical practices, lack of knowledge about health care systems), understanding and treating somatization in multicultural settings is particularly challenging (Perron and Hudelson, 2006; Bäärnhielm, 2012; Dastjerdi, 2012). Based on the available literature, there is a clear need for better access to healthcare services for immigrants that is both culturally and linguistically appropriate and, as well, affordable for low-income individuals (Radl-Karimi et al., 2018). Clinically, depending on the country of origin, health care professionals should be aware of the immigrant patients' tendency to somatize psychological distress and of their ascriptions of meaning of symptoms within a multicultural milieu. Pre-migration, migration, and post-migration experiences all include risk factors for mental health. In this regard, the complexity of both the migratory phenomenon and acculturative stress, with their potentially traumatic burden, should be considered.

The present review supports the need to determine the psychological processes and socioeconomic factors that may increase the tendency to somatize in individuals with migratory backgrounds. From a clinical perspective, it seems essential to identify those subgroups at higher somatization risk through their social and psychological characteristics. Clinical management should include efforts to address inherent emotional distress, as may be generated through their migratory experience. Further, special attention should be paid to the social, cultural and linguistic issues that can pose additional obstacles in the assessment and treatment phases and in the development of a therapeutic alliance with the patient.

## Author Contributions

All authors participated in the concept and writing of this manuscript. All authors approved the final version of the manuscript.

### Conflict of Interest Statement

The authors declare that the research was conducted in the absence of any commercial or financial relationships that could be construed as a potential conflict of interest.
